# Misdiagnosis of Reactive Lymphadenopathy Remotely After COVID-19 Vaccination: A Case Report and Literature Review

**DOI:** 10.3389/fimmu.2022.875637

**Published:** 2022-06-23

**Authors:** Qian Yu, Wei Jiang, Ni Chen, Jia Li, Xiaohui Wang, Maoping Li, Dong Wang, Lan Jiang

**Affiliations:** ^1^ Department of Ultrasound, First Affiliated Hospital of Chongqing Medical University, Chongqing, China; ^2^ Department of Orthopaedics, First Affiliated Hospital of Chongqing Medical University, Chongqing, China; ^3^ Department of Radiology, The First Affiliated Hospital, Chongqing Medical University, Chongqing, China; ^4^ Department of Respiratory and Critical Care Medicine, First Affiliated Hospital of Chongqing Medical University, Chongqing, China

**Keywords:** COVID-19, vaccine, lymphadenopathy, axillary lymph nodes, ultrasonography, contrast-enhanced ultrasonography, side effect

## Introduction

Globally, large-scale COVID-19 vaccination programs are in progress to control the severe acute respiratory syndrome coronavirus 2 (SARS-CoV-2) pandemic (1). As of February 10, 2022, 10.3 billion doses of the vaccines have been administered globally (2). Reactive hyperplasia of the ipsilateral axillary lymph nodes is a side effect of vaccination (3), which has been reported in 0.3% of the participants in the clinical trial of Pfizer (4, 5). Additionally, it has been reported to be rare in the trials of Moderna, Novavax, Sinovac, Johnson & Johnson, and AstraZeneca vaccines (6–10). In reality, the rate is likely to be higher. The Centers for Disease Control and Prevention of the United States (CDC) have reported 11.6 and 16.0% of axillary swelling or tenderness after receiving the first and second doses of Moderna, respectively (11). The frequency of imaging-detected lymphadenopathy ranged between 14.5 and 53% (12). This side effect is a frequent finding after COVID-19 vaccination.

Herein we present a misdiagnosed case of remote lymphadenopathy after receiving the CoronaVac vaccine from Sinovac. We highlight its prolonged course, discuss the clinical findings and imaging features, and analyze our misdiagnosis in combination with a relevant literature review.

## Case Description

A 34-year-old woman presented with left axillary pain for a week and transient fever (38.6°C) for a day. She denied a medical history of allergic disease, tuberculosis, past malignant tumors, recent infection, trauma, specific medication history, and travel or social history. She received the first and second doses of CoronaVac 5 and 4 months ago, respectively, with both doses delivered to the left deltoid muscle. The possibility of side effects was neglected, as the detection exceeded the expected time interval for an adverse reaction to the vaccine. Physical examination revealed left axillary swelling and tenderness with no localized skin or soft tissue lesions, particularly on the head, neck, chest, or left arm.

**Figure 1:**
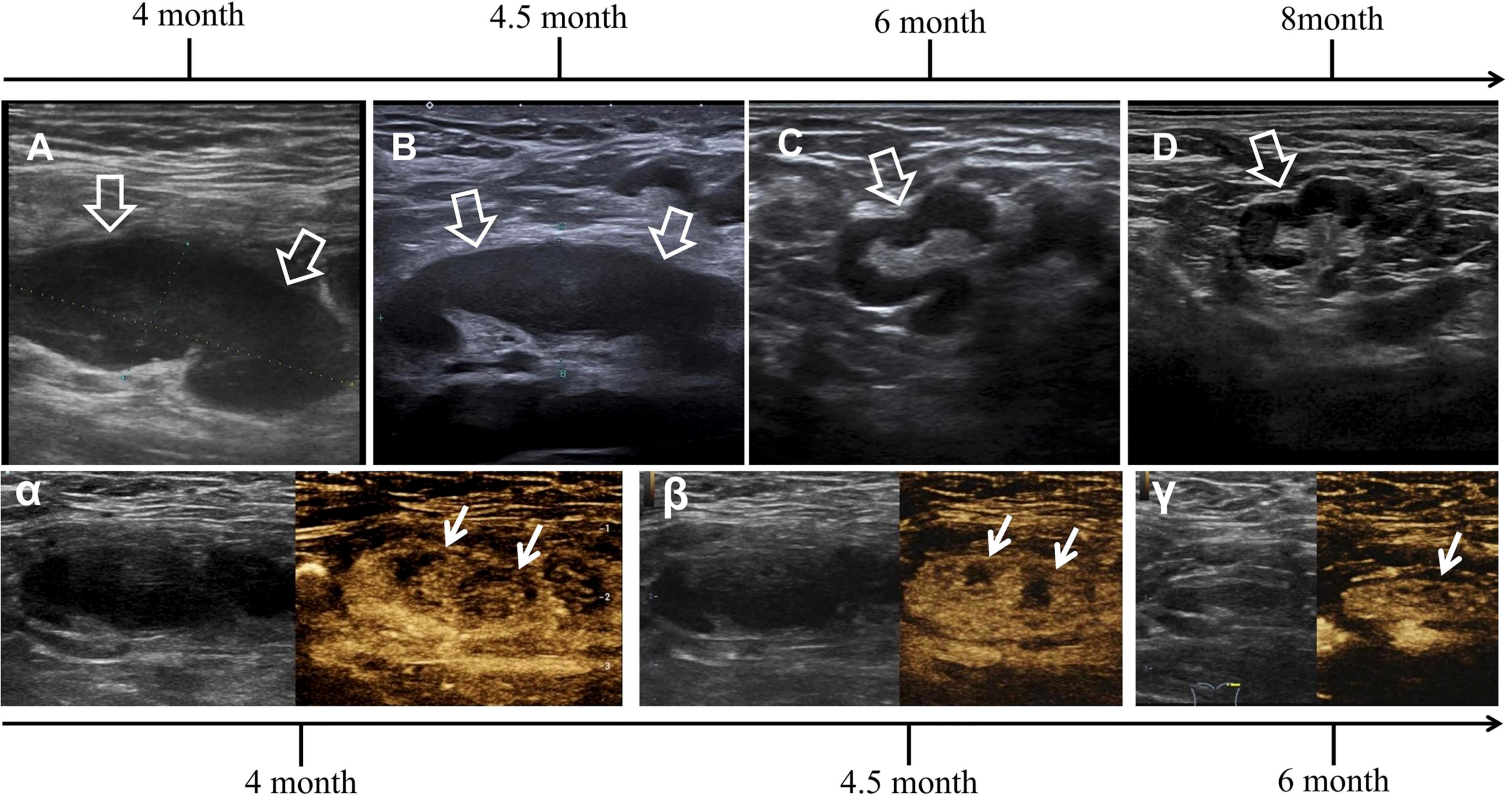
Successive ultrasonography (US) and contrast-enhanced ultrasonography (CEUS) images of the same largest lymph node, with dynamic changes during progression and regression. **(A)** The initial US showed the lymph node was in deep position, flat oval in shape, 40 mm on the long axis and 15 mm on the short axis, with a long/short ratio of >2. The lymphatic cortex was notably thickened to 12 mm and presented as a homogeneous hypoechoic area with a visible lymphatic hilum. **(B)** The second US showed indistinctive decrease in cortical thickness and the same lymph node size from the previous examination. **(C)** The third US in the follow-up showed a distinctive decrease in lymph node size and cortical thickness having irregular shape. **(D)** The last US showed normalized lymph node (indicated by hollow arrows). **(α)** The initial diagnosis of CEUS was based on centripetal perfusion enhancement in the asynchronous type, with a notable area in the deviated center showing hypoperfusion, covering half of the area of the lymph node. **(β)** The second CEUS showed distinctive decrease in hypoperfusion area. **(γ)** The third CEUS in the follow-up showed normalized enhancement and near invisibility of the hypoperfusion area (indicated by arrows).

**Figure 2:**
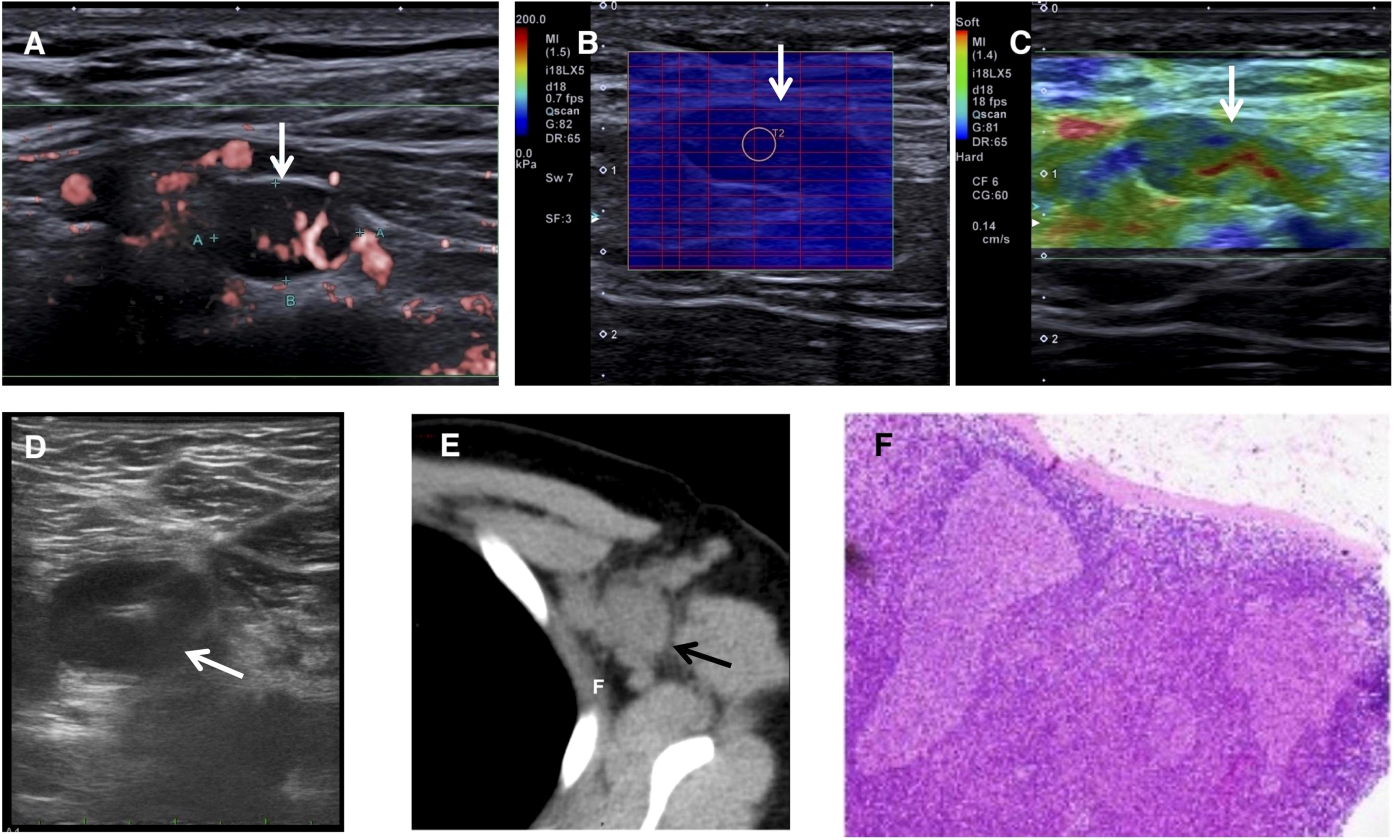
**(A–E)** Images of multiple abnormal lymph nodes (indicated by arrows) at 4 months after vaccination. **(A)** One small abnormal lymph node in the nearly spherical shape, with a long/short ratio of <2, the notably thickened lymphatic cortex with an invisible lymphatic hilum, yet superb microvascular imaging confirmed the blood distribution of hilar type. **(B, C)** Elastography of one of the same abnormal lymph nodes. **(B)** Shear wave elastography demonstrated a modulus of 9.7 kPa. **(C)** Real-time tissue elastography demonstrated the hardness ranging from ‘median’ to ‘soft’. **(D)** US-guided fine-needle aspiration of one superficial lymph node. **(E)** Computed tomography demonstrated the left axillary lymphadenopathy. **(F)** Hematoxylin and eosin staining under 100x magnification showed reactive hyperplasia.

Ultrasonography (US) revealed multiple abnormal left axillary lymph nodes with “alarming” signs (**Figures 1A**, **2A–C**). The relevant diagnostic workup revealed the following: complete blood count (CBC) demonstrated a slight decrease in eosinophils (0.01 × 10^9/^L), the computed tomography (CT) of the head, neck, and chest was normal, and the US of the thyroid, breast, and lymph nodes in other parts of the body and abdomen was also normal (**Figure 2E**). Contrast-enhanced ultrasonography (CEUS) using SonoVue (sulfur hexafluoride microbubbles, Bracco, Netherlands) revealed an internal hypoperfusion area (**Figure 1α**). The sign was misinterpreted as an alarming “necrotic” change and “evidence” of tuberculosis. A US-guided fine-needle aspiration (**Figure 2D**) of one abnormal lymph node (different to the largest one) was performed to confirm the diagnosis; however, the Xpert MTB/RIF assay was negative for the tuberculous gene, the cell smear demonstrated neutrophils and lymphocytes, and the T-cell spot (T-SPOT TB) test and purified protein derivative test were also negative. Thus, tuberculosis and malignancy were excluded, lymphadenopathy was inferred to be bacterial, and treatment with cefaclor (750 mg per os, twice daily for 7 days) was given. The puncture site was fully recovered, but the abnormal lymph nodes never demonstrate a remission.

At 1 week later, the patient presented with transient febrile (40.5°C) again. The US features of the abnormal lymph nodes remained nearly the same, whereas CEUS revealed a noticeable reduction in the hypoperfusion area (**Figures 1B, β**). The laboratory investigations revealed the following parameters: CBC demonstrated decreases in white cell count (3.13 × 10^9^/L), lymphocyte count (0.59 × 10^9^/L), hemoglobin (113 g/L), and hematocrit value (33.8%); the peripheral smear showed normal erythrocyte and leucocyte morphology; the coagulation function showed an increase in D-dimer concentration (1.55 mg/L), fibrinogen (3.92 g/L), and prothrombin time (14.6 s); the liver function test showed an increase in lactic dehydrogenase (762 U/L); the inflammatory biomarkers of procalcitonin (PCT, 0.13 ng/ml), serum ferritin (SF, 354.5 ng/ml), and erythrocyte sedimentation rate (ESR, 69 mm/h) were increased, whereas C-reactive protein and the rheumatoid factors were normal. Immunological tests exhibited negative values for specific infections, including serum IgM antibody titers against influenza virus A/B, parainfluenza virus, respiratory syncytial virus, Epstein–Barr virus, adenovirus, legionella pneumophila, *Mycoplasma pneumoniae*, *Chlamydia pneumoniae*, *Rickettsia*, IgG against hepatitis virus C, syphilis, and HIV antibodies. The quantifications of serum hepatitis B surface antigen (0.00 IU/ml), blood cytomegalovirus DNA (<1.0 × 10^3^), blood Epstein–Barr virus DNA (<1.0 × 10^3^), and serum fungus (1, 3)-β-D glucan (<10 pg/ml) were negative. The aerobic and anaerobic blood cultures were negative for pathogens. The nasopharyngeal swab for the SARS-CoV-2 nucleic acid PCR test was negative for the *ORFlab* and *N* genes. The suspicion of infected lymphadenitis was essentially excluded, and the only remaining concern was histiocytic necrotizing lymphadenitis.

**Figure 3:**
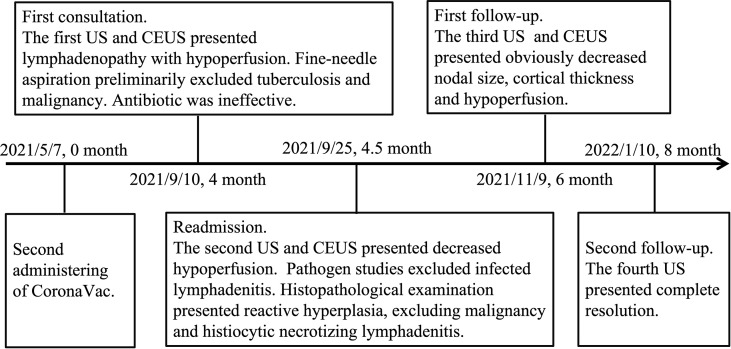
Timeline of diagnosis, interventions, and prognosis. US, ultrasonography; CEUS, contrast-enhanced ultrasonography.

The patient was anxious due to the prolonged diagnostic course and requested a histopathological examination. Macroscopically, the resected abnormal lymph node was soft and yellow-grayish; the microscopy revealed nonspecific reactive hyperplasia (**Figure 2F**), and the immunohistochemistry was negative for tumors. All possible concerns were ruled out except for the idea that vaccination history was reconsidered as the cause. All medical interventions were suspended. In the follow-up at 6 months after vaccination, US demonstrated a notable decrease in the size and cortical thickness of the largest lymph node; the previous hypoperfusion area shown on CEUS was hardly visible (**Figures 1C, γ**). Her laboratory tests of CBC and inflammatory markers were normal. The US indicated complete resolution on the second follow-up at 8 months after vaccination (**Figure 1D**). The lymphadenopathy was finally attributed to COVID-19 vaccine side effects based on clinical, laboratory, imaging, and histopathological findings, and this was confirmed in the prognosis. The timeline of diagnosis, interventions, and prognosis for this case is shown in **Figure 3**.

## Discussion

### Duration

A prolonged course most characterized the presented case of remote reactive lymphadenopathy after COVID-19 vaccination. The symptoms and signs were noticed 4 months after receiving the second dose of CoronaVac, after which the patient improved in 6 months and recovered in 8 months. To our knowledge, no cases of such a remote nature have been previously reported.

In CDC reports, lymphadenopathy occurred within 2 to 4 days after vaccination with Moderna and lasted for 1 to 2 days (11), and the duration in radiologic observational reports was longer (between 4 days and 10 weeks) (12–18). However, most of these cases were retrospective studies in the patients who recently received the COVID-19 vaccine and underwent positron emission tomography (PET). Therefore, there could be selection bias, short observational periods, and lack of reports involving other imaging modalities. This case may be an exception due to individual differences. Nonetheless, the existence of such a prolonged course challenged the current perception about the duration of this side effect.

### Guidelines

Based on the abovementioned observations, current guidelines emphasize the timing for imaging after COVID-19 vaccination. A multidisciplinary expert panel recommended the postponement of imaging for at least 6 weeks after completion of the vaccination (19). The Breast Imaging Society also recommended a scheduling exam conducted at 4–6 weeks following the second dose for screening and a short-term follow-up examination at 4–12 weeks for ipsilateral axillary adenopathy patients who received the COVID-19 vaccine within 4 weeks (20). The recommended timing is practical; however, it cannot encompass exceptional situations, and a cautious approach is needed when facing lymphadenopathy potentially related to vaccines, even in unexpected situations.

### Analysis of Misdiagnosis

This lymphadenopathy case was particularly unusual because the detection occurred outside of the expected time interval with the presence of other clinical findings, of which we were not aware. In hindsight, most of the diagnostic interventions were unnecessary, which resulted in physical and psychological burdens on the patient. Additionally, the misinterpretation of hypoperfusion on CEUS as “perfusion defect” supported the misdiagnosis, as it often suggests “necrosis” of tuberculous or malignant lymphadenopathy (21). Therefore, we must emphasize the importance of rational and subtle image interpretations to avoid both over- and underdiagnosis.

A limitation of this case was the lack of evidence of nucleic acid elements or SARS-CoV-2 antigen on PCR, western blot, or immunohistochemistry tests, which was mainly due to the misdiagnosis that resulted in all of the chosen tests being conducted to verify inflammation or tumors. We propose that biopsy of needle aspiration for PCR or western blot testing would be practical for future diagnostic quandaries that need genetic verification. Additionally, activated lymphocytes should also be examined as an important indicator.

### Imaging Modalities

An accurate diagnosis of lymphadenopathy after vaccination is important. In this case report, we discussed the diagnostic perplexity in an individual without combined medical conditions, whereas reports have also focused on cancer patients (12, 13, 15, 22). A modeling study suggested a large proportion of missed cancer diagnoses due to the pandemic (23). Therefore, subtle imaging interpretation is crucial in patient management.

Concerning imaging-detected reactive lymphadenopathy after COVID-19 vaccination, the majority of cases were identified on PET scans, mainly during cancer surveillance. This condition normally involved transient 18-fluorine-fluorodeoxyglucose (^18^F-FDG) uptake in ipsilateral lymph nodes, ranging from intense to gradually regressed after administration (13, 19, 22, 24–26). However, the maximal standardized uptake value (SUVmax) should not be solely used to differentiate between benign and malignant lymph nodes (14). There were attempts to improve the diagnostic rate by comparing the SUVmax of the ipsilateral lymph nodes to the contralateral lymph nodes (27) as well as the increased uptake in the deltoid muscle (28, 29) or using different tracers (30). PET is the most sensitive imaging modality for differentiating lymphadenopathy, whereas the radioactive nature and high cost are limitations.

Few of the cases were detected *via* magnetic resonance imaging (MRI) (31, 32), CT (15), and mammography (33). These techniques are common diagnostic workups that provide relevant information, especially in the case of breast or lung cancers which often involve axillary lymph node metastasis. They provide good overall observations but relatively few morphological details of abnormal lymph nodes compared to the US (34). Additionally, they cannot provide metabolic features compared to PET (35).

### Role of US

The US has a high diagnostic value in the screening, evaluation, and follow-up of lymphadenopathy (36); it presents high-resolution images of superficial lymph nodes with subtle morphological details that CT or MRI may not have been able to assess (37). We reviewed the full-text accessible literature on US-detected reactive lymphadenopathy after COVID-19 vaccination with a complete vaccination history and explicit image description. The US findings are shown in **Table 1** (34, 38–46). In general, the size, shape, cortex-hilum structure, vascularity patterns, and stiffness of lymph node were essential signs to consider. Rational judgments should be made based on the combined information. It should be noted that there were “alarming” signs mentioned, such as spherical shape, thickened lymphatic cortex, hilum absence, and peripheral vascularity, which have often been observed in malignant or specifically infected lymph nodes (37).

**Table 1:** Ultrasonographic features of 10 published articles on COVID-19 vaccine-related lymphadenopathy (only full-text accessible articles between January 1 and December, 2021 with complete vaccination history and US image description were included).

First author	Interval between US and vaccine	Sum of cases (*N*)	Subclinical ratio	Sum of abnormal lymph nodes in cases (*N*)		Maximum length of LAD (mm)	Maximum thickness of cortex (mm)	Nearly sphere or L/S ratio <2 (*N*)	Hilum		Vascularity patterns			Outcome
				*n* > 3	*n* < 3				Present (*N*)	Absent (*N*)	Hilar (*N*)	Peripheral (N)	Mixed (*N*)	
Washington (38)	12 days	1	–	1	–	31	NA	NA	1	–	NA	NA	NA	Resolved
Granta (39)	1 to 2 days	18	50%	7	11	16	NA	*n* = 12	*n* = 51	*n* = 4	NA	NA	NA	NA
D’Auria (34)	2–8 days	6	1%	6	–	26	6	3	3	3	5	1	–	Reduced
Faermann (40)	1–38 days	125	98%	NA	NA	48	15	NA	NA	NA	NA	NA	NA	Reduced
Mehta (41)	5–13 days	4	100%	2	1	27	7	NA	4	–	3	–	1	NA
Cocco (42)	1–16 days	24	46%	4	20	28	NA	6	16	8	10	1	13	Resolved
Placke (43)	<6 weeks	8	100%	8	–	16	NA	1	2	–	–	–	8	PA
Cristina (44)	1–7 days	91	NA	91	–	Mean: 24	Mean: 4.6	NA	91	–	NA	NA	NA	Resolved
Igual-Rouilleault (45)	20–38 days	10	NA	NA	NA	Thickness: 23	NA	NA	10	–	NA	NA	NA	Resolved: 5 PA: 5
Tsumura (46)	3 weeks	1	100%	NA	NA	35	NA	NA	1	–	–	1	–	NA

N, number of cases; n, number of abnormal lymph nodes; mm, millimeter; L/S, long/short ratio; NA, not available; PA, pathologically verified; -, none.

In this case, the patient underwent 4 successive US examinations that demonstrated dynamic changes during progression and regression. Initially, the US showed increases in the number and size of abnormal lymph nodes, with notably thickened lymphatic cortex (**Figures 1A**, **2A–C**). Regarding the progression of the largest lymph node, the second US showed an indistinctive decrease in cortical thickness, whereas the nodal size remained almost the same. Both size and cortical thickness markedly decreased and eventually resolved at the follow-up examination (**Figures 1A–D**). Based on these trends, we supposed that the thickened cortex might be related to nodal hyperplasia, and it began to subside in the early stage of nodal regression. The decrease in cortical thickness in the US may be an early imaging sign of improvement that should be considered.

### CEUS Findings

To our knowledge, this case was the first to report the CEUS findings of reactive lymphadenopathy after COVID-19 vaccination. The patient underwent 3 successive CEUS scans that also demonstrated dynamic changes. Initially, CEUS demonstrated hypoperfusion in the deviated center of the largest lymph node. It was markedly narrowed in the second CEUS and became nearly invisible in the third exam (**Figures 1** **α**–**γ**). In hindsight, we noted that the initial hypoperfusion area overlapped with the most thickened cortex; moreover, it regressed even earlier than the cortex attenuation. A decrease of hypoperfusion may be an even earlier sign of improvement. We speculated that the COVID-19 vaccine might stimulate immune cells in the nodal cortex, which leads to excessive pressure on tissue microcirculation. The insufficiency of perfusion was represented as filling insufficiency on CEUS. However, this was merely a conjecture regarding pathogenesis without the support of systematic research, and further work is required to elucidate this mechanism.

### Concomitant Manifestations

In this case, accompanying manifestations interfered with the diagnosis, including transient fever, decreased white cell and lymphocyte counts, increased inflammatory markers (ESR, PCT, and SF), and abnormal coagulation function. A previous study reported a decrease in lymphocytes after COVID-19 vaccination (26), and elevated PCT and CRP were independent risk factors for death in patients with COVID-19 (47). As all tests for specific infections were negative, and the patient fully recovered without medical intervention, we retrospectively supposed that these manifestations were also vaccine reactions. However, local and systemic inflammatory reactions after COVID-19 vaccination should be transient, and further studies are needed to describe remote reactions after COVID-19 vaccination. Additionally, the patient presented with mild anemia in the second CBC, possibly due to malnutrition because of anxiety.

### Vaccine Type

Concerning the type of COVID-19 vaccine that causes reactive lymphadenopathy, there have been more than 2,000 reported cases after mRNA vaccines and 14 reported cases after adenoviral vectored vaccines (12, 22), yet there have been no specific case reports related to protein subunit vaccines. In theory, regardless of the type, all COVID-19 vaccines might cause reactive lymphadenopathy, and this case can serve as a supplement to observational side effect studies.

## Conclusion

Radiologists and clinicians should recognize that reactive lymphadenopathy has become frequently observed in association with the general administration of COVID-19 vaccines. It should be considered a frequent and important differential diagnosis. Rational judgment should be made in the context of vaccination information and subtle imaging interpretations. Herein we propose the existence of a prolonged course of this side effect, the value of US as a diagnostic workup and evaluation, and the first introduction of CEUS through the presented case.

## Data Availability Statement

The original contributions presented in the study are included in the article/supplementary material. Further inquiries can be directed to the corresponding author.

## Ethics Statement

Written informed consent was obtained from the individual for the publication of any potentially identifiable images or data included in this article.

## Author Contributions

QY and JL were major contributors in preparation of the manuscript. LJ interpreted the patient data and drafted the paper. QY did the literature review. WJ drafted the paper. NC and ML performed the US and CEUS. JL, XW, and DW interpreted the patient data. All authors contributed to the article and approved the submitted version.

## Conflict of Interest

The authors declare that the research was conducted in the absence of any commercial or financial relationships that could be construed as a potential conflict of interest.

## Publisher’s Note

All claims expressed in this article are solely those of the authors and do not necessarily represent those of their affiliated organizations, or those of the publisher, the editors and the reviewers. Any product that may be evaluated in this article, or claim that may be made by its manufacturer, is not guaranteed or endorsed by the publisher.
